# Improvement of Prediction Ability for Genomic Selection of Dairy Cattle by Including Dominance Effects

**DOI:** 10.1371/journal.pone.0103934

**Published:** 2014-08-01

**Authors:** Chuanyu Sun, Paul M. VanRaden, John B. Cole, Jeffrey R. O'Connell

**Affiliations:** 1 National Association of Animal Breeders, Columbia, Missouri, United States of America; 2 Animal Genomics and Improvement Laboratory, Agricultural Research Service, United States Department of Agriculture, Beltsville, Maryland, United States of America; 3 University of Maryland School of Medicine, Baltimore, Maryland, United States of America; CSIRO, Australia

## Abstract

Dominance may be an important source of non-additive genetic variance for many traits of dairy cattle. However, nearly all prediction models for dairy cattle have included only additive effects because of the limited number of cows with both genotypes and phenotypes. The role of dominance in the Holstein and Jersey breeds was investigated for eight traits: milk, fat, and protein yields; productive life; daughter pregnancy rate; somatic cell score; fat percent and protein percent. Additive and dominance variance components were estimated and then used to estimate additive and dominance effects of single nucleotide polymorphisms (SNPs). The predictive abilities of three models with both additive and dominance effects and a model with additive effects only were assessed using ten-fold cross-validation. One procedure estimated dominance values, and another estimated dominance deviations; calculation of the dominance relationship matrix was different for the two methods. The third approach enlarged the dataset by including cows with genotype probabilities derived using genotyped ancestors. For yield traits, dominance variance accounted for 5 and 7% of total variance for Holsteins and Jerseys, respectively; using dominance deviations resulted in smaller dominance and larger additive variance estimates. For non-yield traits, dominance variances were very small for both breeds. For yield traits, including additive and dominance effects fit the data better than including only additive effects; average correlations between estimated genetic effects and phenotypes showed that prediction accuracy increased when both effects rather than just additive effects were included. No corresponding gains in prediction ability were found for non-yield traits. Including cows with derived genotype probabilities from genotyped ancestors did not improve prediction accuracy. The largest additive effects were located on chromosome 14 near *DGAT1* for yield traits for both breeds; those SNPs also showed the largest dominance effects for fat yield (both breeds) as well as for Holstein milk yield.

## Introduction

Simulations and validation studies using real data have indicated that genomic selection can provide remarkably high accuracy of predicted breeding values (BV) of individuals without their own records or without progeny records [1], [2], which offers the opportunity to select individuals as parents of the next generation accurately at an early stage of life. This technique has become a standard tool in dairy cattle breeding [3] and is rapidly expanding to other agriculturally important species (e.g., poultry [4], pig [5], and plant breeding [6]).

Few studies have attempted to generalize and apply genomic selection models that include non-additive genetic effects with large data sets [7]. Non-additive genetic variation results from interactions between alleles, and the interaction between alleles at the same locus is called dominance. Dominance is an important non-additive genetic effect, and the inclusion of dominance effects in models for the prediction of genomic BV could increase the accuracy of the predictions [8], [9]. However, genotypes and phenotypes for the same individuals must be known to detect allelic interaction. For some traits, the expression is naturally limited to females and estimated BV (EBV) or de-regressed EBV obtained from routine evaluations [10] are used as phenotypes in most applications of genomic selection. Such data allow only the estimation of allele substitution effects, and distinguishing between additive and dominance effects is not possible. The increasing availability of cows with phenotypes and genotypes in the United States now provides an opportunity to investigate models that include dominance effects. Sun et al. [11] estimated dominance variance using only cows that had genotypes and phenotypes for milk yield in the U.S. national database but did not test predictive ability for a model that included a dominance effect.

Although many cows with phenotypes do not have genotypes, their sires and dams or their sires and maternal grandsires (MGS) have genotypes.. The expected genotype probabilities for those cows based can be calculated using genotypes of the ancestors and the allele frequencies in the population. Boysen et al. [12] discovered significant dominance effects for yield traits in dairy cattle by regression of phenotypes on such derived genotype probabilities; however, they did not investigate if model prediction improved when cows with derived genotype probabilities were included in the analysis.

Many statistical models and algorithms have been proposed to predict BV using genome-wide dense markers, which differ in the assumption of distributions of SNP effects [13]. Two models to compute genomic best linear unbiased predictions (BLUP) [1] assume normally distributed SNP effects. They have become popular approaches in practical genomic evaluation because they are simple and have low computational demands, as well as similar performance with variable selection models [3], [14]. One estimates marker effects using random regression on marker genotypes, and genomic BV are calculated as the sum of estimated marker effects (hereafter called SNP-BLUP). The other estimates genomic BV directly using a marker-based relationship matrix (hereafter called GBLUP). These two BLUP models can be easily extended to include dominance effects [15]. However, different sets of dominance coefficients can be derived that can result in different predictions [16].

This study had four goals. First, additive and dominance variance components were estimated using Holstein and Jersey data for eight traits. Second, predictive ability of models that included additive and dominance effects was compared with that of a model that included only additive effects. Third, predictions obtained using different dominance coefficients were compared. Fourth, model prediction was tested by expanding the data set to include cows with genotype probabilities derived based on ancestor genotypes.

## Materials and Methods

### Data

Genotypes were available from the Council on Dairy Cattle Breeding (Reynoldsburg, OH, USA) for Holsteins and Jerseys. Genotypes were from six different SNP arrays: the Bovine3K, BovineLD, BovineSNP50, and BovineHD (Illumina Inc., San Diego, CA), and the GeneSeek Genomic Profiler and GeneSeek Genomic Profiler HD (Neogen Agrigenomics, Lincoln, NE, USA). All genotypes were imputed to a BovineSNP50 basis using findhap.f90 software [17] before estimating genomic BV and dominance effects.

Phenotypic data were yield deviations for milk, fat, and protein; productive life (PL); daughter pregnancy rate (DPR); somatic cell score (SCS), fat percent (fat%) and protein percent (protein%) for first parity. Yield deviations for fat% and protein% were obtained indirectly as (yield deviation of fat% = ((fat mean for base cows+fat yield deviation)/(milk mean for base cows+milk yield deviation) - fat mean for base cows/milk mean for base cows) *100; and a corresponding formula for protein%). The values of trait mean for base cows were 11,839, 432 and 396Kg for Holstein milk, fat and protein, respectively, and corresponding values were 8379, 384 and 298Kg for Jersey breed. DPR is defined as percentage of non-pregnant cows that become pregnant during each 21-day period; a DPR of 1 implies that cows are 1% more likely to become pregnant during that estrus cycle than cows with an evaluation of 0. PL is defined as time in the milking herd before removal by voluntary culling, involuntary culling, or death; credits for each month in milk are obtained from standard lactation curves and then summed across all lactations; diminishing credits within lactation give cows more credit for beginning a new lactation than for continuing to milk in previous lactation; cows get 8 months credit for 305-day first-lactation records, 10 months credit for second lactations, 10.2 months credit for third and later lactations, partial credits for shorter records, and extra credits for longer records.

The data set was divided into three groups. The first set included cows with known genotypes and phenotypes (DATA_C_). The second included cows with phenotypes, but genotype probabilities were calculated from genotyped sire and dam (DATA_S-D_). The third included cows with phenotypes but genotype probabilities were calculated from genotyped sire and MGS (DATA_S-MGS_ [12]).

Tables 1 and 2 listed phenotypic information for each of the data groups and six traits. Fixed effects (age and parity group, herd management group, inbreeding, and heterosis) were first estimated using a multi-trait and multi-breed linear mixed model from the full national data set of phenotype and pedigree information, and then records from first parity were adjusted for fixed effects (age and parity group and herd management group) for the subset of cows that had both phenotypic and genotypic information (Table 1). For yield, fat% and protein% traits, records were available from 30,482 Holstein and 8,321 Jersey cows; for other traits, 14,780 Holstein and 5,492 Jersey PL records, 23,811 Holstein and 7,422 Jersey DPR records, and 30,352 Holstein and 8,292 Jerseys SCS records were available. Yield means (two fixed effect adjustment) were larger for Holsteins than for Jerseys, but Jerseys had better performance for PL and DPR. The mean and standard deviation of inbreeding and heterosis for Holstein were lower than Jersey. The inbreeding effects from multi-trait and multi-breed model were −66.12, −2.47, −1.96, −0.268, −0.072, and 0.004 for milk, fat, protein, PL, DPR and SCS trait, respectively, and corresponding heterosis effects were 172.23, 22.12, 11.29, 0.349, 1.973, and 0.019.

**Table 1:** Phenotypic, inbreeding and heterosis statistics for Holstein and Jersey milk, fat, and protein yields, productive life (PL), daughter pregnancy rate (DPR), somatic cell score (SCS), fat percent (fat%) and protein percent (protern%) based on genotyped cows.

Trait			Milk (kg)	Fat (kg)	Protein (kg)	PL (months)	DPR (%)	SCS	fat%	protein%
HO	Phenotype	N^a^	30,482	30,482	30,482	14,780	23,811	30,352	30,482	30,482
		Mean	10,857	388	337	42.1	24.4	2.77	−0.001	0.061
		SD^b^	1,635	63	46	22.3	17	1.47	0.206	0.087
	inbreeding	Mean	5.66	5.66	5.66	5.53	5.61	5.66	5.66	5.66
		SD	1.65	1.65	1.65	1.67	1.63	1.65	1.65	1.65
		regCoff^c^	−66.12	−2.47	−1.96	−0.268	−0.072	0.004	−	−
	Heterosis	Mean	0.0014	0.0014	0.0014	0.0022	0.0016	0.0014	0.0014	0.0014
		SD	0.0284	0.0284	0.0284	0.0377	0.0308	0.0285	0.0284	0.0284
		regCoff	172.23	22.12	11.29	0.349	1.973	0.019	−	−
JE	Phenotype	N	8,321	8,321	8,321	5,492	7,422	8,292	8,321	8,321
		Mean	8,284	374	302	49.4	30.9	3.08	0.015	0.06
		SD	1,434	63	46	22.6	16.5	1.41	0.289	0.127
	inbreeding	Mean	6.63	6.63	6.63	6.66	6.66	6.64	6.63	6.63
		SD	2.92	2.92	2.92	2.98	2.95	2.92	2.92	2.92
		regCoff	−66.12	−2.47	−1.96	−0.268	−0.072	0.004	−	−
	Heterosis	Mean	0.0112	0.0112	0.0112	0.0127	0.0121	0.0109	0.0112	0.0112
		SD	0.0884	0.0884	0.0884	0.0961	0.0929	0.0865	0.0884	0.0884
		regCoff	172.23	22.12	11.29	0.349	1.973	0.019	−	−

a Number of records,^ b^SD = Standard deviation, ^c^regCoff was the regression coefficient for inbreeding or heterosis, which was estimated based on a multiple-trait and multiple-breed linear mixed model.

**Table 2:** Phenotypic statistics for Holstein and Jersey milk, fat, and protein yields based on cows with genotype probabilities derived using genotyped sire and dam (S-D) or genotyped sire and maternal grandsire (S-MGS).

Genotyped ancestors	Trait	Holstein			Jersey		
		Records (no.)^a^	Mean (kg)	Standard deviation (kg)	Records (no.)^a^	Mean (kg)	Standard deviation (kg)
S-D	Milk	25,926	10,550	1,582	4,896	7,483	1,459
	Fat	25,926	372	61	4,896	333	66
	Protein	25,926	327	44	4,896	273	48
S-MGS	Milk	33,897 (2,278,652)	9,858	750	11,823 (379,713)	6,899	872
	Fat	33,897 (2,278,652)	346	26	11,823 (379,713)	311	34
	Protein	33,897 (2,278,652)	304	22	11,823 (379,713)	253	27

a Total number of daughters in S-MGS group in parentheses.

For non-genotyped cows, whose genotype probabilities were derived using genotyped sires and dams or genotyped sires and MGS (Table 2), records were available from 25,926 Holsteins and 4,896 Jerseys with sire and dam genotypes and from 33,897 Holstein and 11,823 Jersey S-MGS groups. Each sire-MGS pair was required to have ≥20 observations for Holsteins and ≥8 observations for Jerseys, and the S-MGS groups included 2,278,652 Holstein and 379,713 Jersey cows. Based on Tables 1 and 2, means and standard deviations were different for DATA_C_, DATA_S-D_ and DATA_S-MGS_ for yield traits.

Given a specific marker locus with two alleles (A and B), the probabilities of possible genotypes (AA coded as 0, AB coded as 1, and BB coded as 2) for cows were computed as
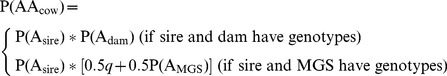





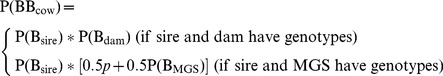
where P(A_sire_), P(A_dam_), and P(A_MGS_) are the probabilities that allele A was transmitted to offspring from sire, dam and MGS, respectively; P(B_sire_), P(B_dam_), and P(B_MGS_) are the probabilities that allele B was transmitted to offspring from sire, dam and MGS, respectively; and population allele frequencies were *q* for A and *p* for B. Then



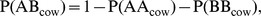



and
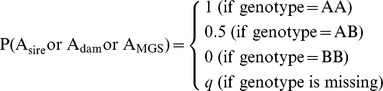



The same approach was used to calculate P(B_sire_ or B_dam_ or B_MGS_).

The DATA_C_ data set was used to estimate variance components and SNP effects (additive and dominance) and to perform ten-fold cross-validation for prediction. Variance estimation and validation were also conducted using the combined data sets (DATA_C_+DATA_S-D_+DATA_S-MGS_). The same testing data sets were used when cross-validation was performed on DATA_C_ only or on the combined data sets.

### Variance Components

Variance components for each trait were estimated using the GBLUP method by including additive or additive and dominance genetic effects; the single-trait linear mixed models used were:
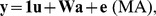





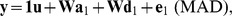





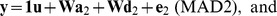





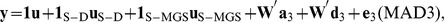
where **y** is a vector of management group deviations for each trait; **u**, **u**_S-D_, and **u**_S-MGS_ are the intercepts; **a**, **a**_1_, **a**_2_, and **a**_3_ are vectors of additive effects for animals; **d**_1_, **d**_2_, and **d**_3_ are vectors of dominance effects; **e**, **e**_1_, **e**_2_, and **e**_3_ are the vectors of random residuals for animals; **1** is a vector with elements of 1, and **1**_S-D_ and **1**_S-MGS_ are vectors with elements of 1 for DATA_S-D_ and DATA_S-MGS_, respectively, and 0 for other records. Each animal had a single record; therefore, 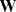 and 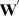 were identity matrices.

Then, 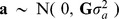, 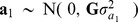, 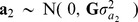, 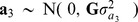, 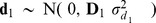, 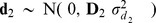, 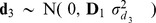, 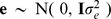, 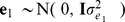, 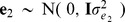, and 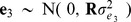, where **G** and **D**_1_ (or **D**_2_ **)** are additive and dominance genomic relationship matrices, respectively; 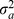, 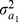, 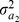 and 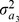 are additive variances; 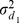, 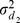, and 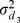 are dominance variances; 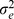, 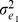, 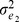, and 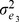 are residual variances, and **R** is the coefficient matrix for error variance:
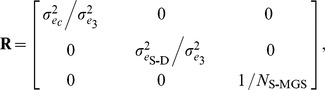
where 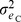 is the residual variance for genotyped cows, 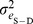 is residual variance for cows with genotype probabilities derived from genotyped sire and dam, and *N*_S-MGS_ is the number of daughters for each sire-MGS pair. The **G,** **D**_1_, and **D**_2_ were constructed based on information from genome-wide markers [1], [9], [15], [16]: 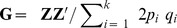, 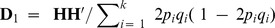, and 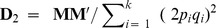, where *k* is the total number of SNPs; **Z** is a centered genotype matrix with each *z* is a genotype code (0, 1, or 2) minus 2*p_i_*; *p_i_* is the frequency of the second of two alleles at locus *i*; *q_i_* is the frequency of the first allele at locus *i*; the elements of **H** equal 0−2*p_i_q_i_* for homozygous alleles and 1−2*p_i_q_i_* for heterozygous alleles; and the elements of **M** equal 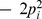, 2*p_i_q_i_*, and 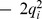 for genotype codes 0, 1, and 2, respectively. The differences between MAD and MAD2 were explained and investigates in detail in a previous study [16].

Variance components were estimated using average-information restricted maximum likelihood (AI-REML) [18] as implemented in MMAP (mixed models analysis for pedigrees and populations) software [19], [20]. The MMAP software incorporates the Intel Math Kernel Library [21] for optimized parallel matrix algebra and likelihood calculation.

### SNP Effects

The additive and dominance effects for each SNP were estimated using the SNP-BLUP method with the variance components described previously. Using the MAD model as an example, the mixed model equation for estimating each SNP effect was.



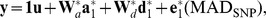
where 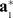
and 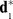
are vectors of additive and dominance effects, respectively, for SNP; 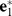 is residual variances; 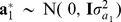, 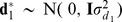 and 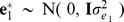; 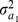
and 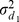
are total additive and dominance variances, and need to divide 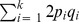
and 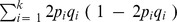, respectively, for each marker; 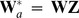, 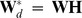, and 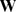, 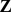 and 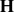 are the same as defined before. Since
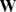
is the identity matrix in our case, the mixed model equations for MAD_SNP_ are
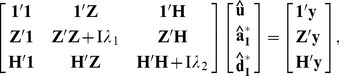
(1)where 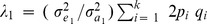 and 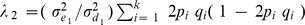 [1]. Similarly, the SNP-BLUP versions for the MA, MAD2, and MAD3 models can be built easily and defined as MA_SNP_, MAD2_SNP_, and MAD3_SNP_, respectively.

Solutions for small populations can be obtained directly by building the mixed model equations shown in (1) and inverting the left-hand side. The MA_SNP_, MAD_SNP_, and MAD2_SNP_ models used data only from DATA_C_, and equations were solved by the inversion method. However, the MAD3_SNP_ model used data from all three data sets (DATA_C_, DATA_S-D_, and DATA_S-MGS_). Because some cow genotypes were probabilities and required >1 character for storage, calculations for 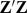, 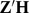, and 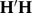 in (1) required much more time, memory, and disk space. An iteration-based program was developed to solve MAD3_SNP_ for big data. A blend of first- and second-order Jacobi iteration was implemented with two relaxation factors [1]. Manhattan plots of the additive and dominance effects were created using ggplot2 [22], version 0.9.2, and R-2.15.1 [23].

### Model Validation

Goodness-of-fit for each model was evaluated using likelihoods based on the whole data set as well as correlations between predicted BV and phenotypes in the training data. The superiority of model MAD and MAD2 over MA was tested using a likelihood ratio test. Cross-validation was used to measure prediction accuracy, with the data set randomly divided into ten approximately equal portions. Nine of the portions were used in turn for training the models to estimate SNP effects, and the remaining portion was used for testing prediction accuracy. The predictive ability of the model was evaluated by comparing predictions and phenotypes of animals in the testing data set and was measured as the correlation between predicted genetic values and phenotypes. Predictions of additive genetic effect (BV) and total genetic value (defined as the sum of additive and dominance effects in the model) were both evaluated. Paired two-sample t-tests were used to test correlations for differences.

## Results

### Variance Component Estimation and Heritability

Table 3 shows estimates of variance components and heritabilities using the MA, MAD, and MAD2 models for each of the eight traits; MAD3 was only applied to yield traits. For both Holsteins and Jersey yield traits, MAD had lower additive heritabilities and higher dominance heritabilities than MAD2, but the sum of additive and dominance variances were similar for both models. The MAD2 additive heritabilities were much closer than MAD additive heritabilities to MA heritabilities. Based on MAD and MAD2, dominance variance accounted for 5% and slightly less than 4%, respectively, of phenotypic variance for Holstein yield traits and 7% and 5.5% of Jersey yield traits. Additive heritability estimates from MAD3 were lower than from MAD and MAD2; MAD3 dominance variances were similar to those from MAD2 for Jerseys but smaller for Holsteins. Dominance variances from MAD and MAD2 were very small for DPR and SCS regardless of breed, especially for DPR. Dominance variance for PL was larger for Jerseys than for Holsteins. Fat% and protein% had high additive but low dominance heritabilities.

**Table 3:** Holstein and Jersey estimated variance components and heritabilities for milk, fat, and protein yields, productive life (PL), daughter pregnancy rate (DPR), somatic cell score (SCS), fat percent (fat%) and protein percent (protein%) using four different models.

Breed	Model^a^	Var^b^	Milk (kg^2^)	Fat (kg^2^)	Protein (kg^2^)	PL (months^2^)	DPR (%^2^)	SCS	Fat %	protein%
HO	MA	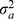	762,478	1,002	449	21.2	16.1	0.19	0.021	0.0038
		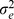	1,885,769	2,961	1,579	476.6	273.3	1.98	0.023	0.0039
		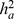	0.288	0.253	0.221	0.043	0.056	0.087	0.468	0.492
	MAD	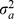	712,905	920	409	21.1	16.6	0.18	0.02	0.0038
		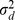	134,815	200	108	0.24	0.001	0.02	0.0005	0.0001
		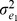	1,788,979	2,824	1,501	476.4	274.6	1.96	0.023	0.0039
		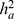	0.27	0.233	0.202	0.042	0.057	0.084	0.464	0.487
		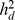	0.051	0.051	0.053	0	0	0.01	0.011	0.013
	MAD2	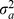	750,379	985	437	20.9	16.1	0.19	0.021	0.0038
		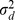	96,834	136	79	2.4	0.002	0.02	0.0003	0.00006
		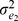	1,787,699	2,827	1,499	474.4	274.1	1.96	0.023	0.0039
		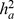	0.285	0.25	0.217	0.042	0.056	0.087	0.468	0.492
		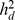	0.037	0.034	0.039	0.005	0	0.01	0.006	0.008
	MAD3	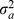	831,607	999	548	–	–	–	–	–
		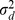	92,019	118	73	–	–	–	–	–
		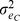	1,848,844	2,925	1,543	–	–	–	–	–
		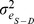	2,218,018	3,332	1,778	–	–	–	–	–
		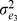	2,950,420	3,840	2,328	–	–	–	–	–
		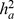	0.215	0.202	0.186	–	–	–	–	–
		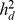	0.024	0.024	0.025	–	–	–	–	–
JE	MA	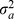	735,064	869	552	36.3	9.3	0.21	0.045	0.009
		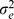	1,354,587	3,050	1,585	477.9	263.6	1.82	0.038	0.0068
		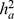	0.352	0.222	0.258	0.071	0.034	0.102	0.542	0.567
	MAD	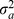	668,822	747	490	29.2	8.1	0.2	0.044	0.0088
		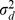	144,809	279	149	19.6	3.2	0.02	0.0007	0.0005
		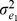	1,262,034	2,871	1,488	465	261.5	1.8	0.037	0.0065
		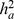	0.322	0.192	0.23	0.057	0.03	0.098	0.54	0.556
		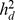	0.07	0.072	0.07	0.038	0.012	0.012	0.009	0.03
	MAD2	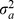	713,008	834	534	35.8	9.3	0.21	0.045	0.0089
		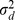	112,263	215	119	12.2	0.1	0.02	0.0005	0.0003
		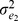	1,250,286	2,849	1,473	466.2	263.5	1.8	0.037	0.0065
		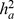	0.344	0.214	0.251	0.07	0.034	0.102	0.543	0.567
		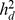	0.054	0.055	0.056	0.024	4E-04	0.01	0.006	0.020
	MAD3	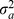	827,677	955	618	–	–	–	–	–
		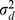	157,350	306	161	–	–	–	–	–
		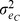	1,307,028	2,919	1,537	–	–	–	–	–
		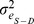	1,848,349	3,826	2,051	–	–	–	–	–
		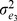	2,068,021	3,985	2,217	–	–	–	–	–
		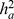	0.271	0.182	0.206	–	–	–	–	–
		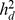	0.052	0.058	0.054	–	–	–	–	–

HO = Holstein; JE = Jersey.

a MA = only additive effects included; MAD = additive and dominance (values) effects included; MAD2 = additive and dominance (deviations) effects included; and MAD3 = additive and dominance effects included as well as cows with genotype probabilities derived using genotyped ancestors.

b Var = variance components; 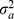 = additive variance, 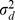 = dominance variance, 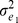 = residual variance for MAD, 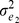 = residual variance for MAD2, 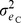 = residual variance for genotyped cows, 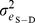 = residual variance for cows with genotype probabilities derived from genotyped sire and dam, 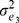 = residual variance for MAD3, 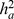 = additive heritability, and 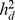 = dominance heritability.

### Model Goodness-of-Fit

Measures of goodness-of-fit based on likelihood ratio tests are in Table 4. For Holstein and Jersey yield traits, the likelihood ratio test showed that MAD and MAD2 fit the data significantly (*P*<0.0001) better than did MA. For PL, DPR, and SCS, the −2 log likelihoods were similar for MA, MAD, and MAD2. The model including dominance also fit the data better than MA for protein% (both breed) and fat% of Holstein. The number of animals in MAD3 was different from that for MA, MAD, and MAD2; therefore, the likelihood for MAD3 was not comparable with that for other models.

**Table 4:** Holstein and Jersey likelihood statistics (−2 log likelihood, *P*-value of *χ* ^2 ^test^b^ using likelihood ratio) for milk, fat, and protein yields, productive life (PL), daughter pregnancy rate (DPR), somatic cell score (SCS), fat percent (fat%) and protein percent (protein%) using three different models.

Breed	Likelihood statistic	Model^a^	Milk	Fat	Protein	PL	DPR	SCS	fat%	protein%
HO	-2Log	MA	523,875	326,549	306,908	106,438	158,369	52,811	-76,286	-130,192
		MAD	523,689	326,407	306,728	106,438	158,370	52,807	-76,294	-130,204
		MAD2	523,687	326,416	306,723	106,437	158,370	52,805	-76,292	-130,202
	*P*-value	MA, MAD	1.19E-42	4.86E-33	2.42E-41	1	1	0.023	0.0047	0.0005
		MA, MAD2	4.34E-43	4.52E-31	1.96E-42	0.159	1	0.007	0.0143	0.0016
JE	-2Log	MA	141,040	89,552	84,317	39,698	49,014	13,892	-15,500	-29,456
		MAD	141,005	89,519	84,284	39,694	49,013	13,891	-15,502	-29,464
		MAD2	140,998	89,514	84,276	39,695	49,014	13,891	-15,502	-29,464
	*P*-value	MA, MAD	1.65E-09	4.61E-09	4.61E-09	0.023	0.159	0.159	0.1573	0.0047
		MA, MAD2	4.56E-11	3.54E-10	7.61E-11	0.042	1	0.159	0.1573	0.0047

a MA = only additive effects included; MAD = additive and dominance (values) effects included; and MAD2 = additive and dominance (deviations) effects included.

b
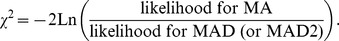

Average correlations between estimated genetic effects and phenotypes in training data for ten-fold cross-validation (Table 5) also indicated model goodness-of-fit. Correlations between total genetic effects (additive for MA and additive plus dominance for MAD, MAD2, and MAD3) and phenotypes were higher for MAD and MAD2 than for MA for all Holstein and Jersey traits. For MAD3, correlations between total genetic effects and phenotypes were higher than for MA but lower than for MAD and MAD2; correlations between additive effects and phenotypes were lowest. The standard deviations of correlations were from 0.001 to 0.003 for Holstein, and from 0.001 to 0.005 for Jersey, across different traits; PL and milk had the largest and smallest standard deviation, respectively. This was true using MAD, MAD2 or MAD3. Because the yield deviations of fat% and protein% were derived from yield traits and their dominance variances were small, the ten-fold cross-validation was not carried out on fat% and protein%.

**Table 5:** Holstein and Jersey average correlations between estimated genetic effects and phenotypes for milk, fat, and protein yields, productive life (PL), daughter pregnancy rate (DPR), and somatic cell score (SCS) from training data for ten-fold cross-validation for four models.

Breed	Model^a^	Genetic effect	Milk	Fat	Protein	PL	DPR	SCS
Holstein	MA	Additive	0.601	0.574	0.557	0.367	0.352	0.389
	MAD	Additive	0.614	0.584	0.564	0.367	0.355	0.394
		Additive+dominance	0.662	0.636	0.617	0.417	0.363	0.419
	MAD2	Additive	0.615	0.586	0.564	0.366	0.355	0.395
		Additive+dominance	0.665	0.636	0.622	0.431	0.370	0.424
	MAD3	Additive	0.589	0.556	0.538	–	–	–
		Additive+dominance	0.642	0.605	0.597	–	–	–
Jersey	MA	Additive	0.681	0.604	0.623	0.471	0.367	0.489
	MAD	Additive	0.693	0.612	0.633	0.473	0.365	0.493
		Additive+dominance	0.763	0.694	0.716	0.604	0.440	0.527
	MAD2	Additive	0.694	0.612	0.635	0.473	0.367	0.493
		Additive+dominance	0.764	0.703	0.722	0.592	0.371	0.534
	MAD3	Additive	0.630	0.540	0.542	–	–	–
		Additive+dominance	0.743	0.660	0.672	–	–	–

a MA = only additive effects included; MAD = additive and dominance (values) effects included; MAD2 = additive and dominance (deviations) effects included; and MAD3 = additive and dominance effects included as well as cows with genotype probabilities derived using genotyped ancestors.

### Prediction Accuracy

Predictive ability for Holstein and Jersey yield traits was better for MAD and MAD2 than for MA based on correlations from testing data used in the ten-fold cross-validation (Table 6). For MAD and MAD2, correlations were higher between phenotype and total genetic effects than between phenotype and additive-only effects for yield traits, and both MAD and MAD2 correlations were higher than those between phenotype and additive effect from MA. The differences between correlations from MAD or MAD2 and that from MA were statistically significant for Holstein yield traits and SCS (*P*<0.005) and Jersey yield traits (*P*<0.001). However, for Jersey PL, DPR, and SCS as well as Holstein PL and DPR, correlations from MA, MAD, and MAD2 from testing data were almost the same and did not differ statistically (*P*>0.2). Jersey correlations from testing data were lower than Holstein correlations except for PL. By enlarging the data set, MAD3 did not provide better prediction for either Holsteins or Jerseys. The standard deviation of correlations from ten-fold cross-validation ranged from 0.017 to 0.024 on different traits for Holstein, and from 0.018 to 0.043 for Jersey; yield traits had lower standard deviation than other traits.

**Table 6:** Holstein and Jersey average correlations between estimated genetic effects and phenotypes for milk, fat, and protein yields, productive life (PL), daughter pregnancy rate (DPR), and somatic cell score (SCS) from testing data for ten-fold cross-validation for four models as well as *P*-values from paired t-tests based on differences between model correlations.

Breed	Model^a^/*P*-value	Genetic effect	Milk	Fat	Protein	PL	DPR	SCS
Holstein	MA	Additive	0.440	0.409	0.399	0.108	0.158	0.198
	MAD	Additive	0.452	0.419	0.405	0.108	0.159	0.202
		Additive+dominance	0.458	0.425	0.412	0.108	0.159	0.203
	MAD2	Additive	0.451	0.419	0.405	0.108	0.159	0.202
		Additive+dominance	0.460	0.426	0.415	0.108	0.159	0.203
	MAD3	Additive	0.433	0.388	0.375	–	–	–
		Additive+dominance	0.441	0.396	0.385	–	–	–
	*P*_MA,MAD_	Additive	<0.001	<0.001	<0.001	0.547	0.269	0.003
		Additive+dominance	<0.001	<0.001	<0.001	0.831	0.299	<0.001
	*P*_MA, MAD2_	Additive	<0.001	<0.001	0.002	0.634	0.268	0.003
		Additive+dominance	<0.001	<0.001	<0.001	0.956	0.324	0.001
Jersey	MA	Additive	0.419	0.356	0.356	0.107	0.092	0.170
	MAD	Additive	0.428	0.361	0.361	0.109	0.091	0.169
		Additive+dominance	0.441	0.371	0.373	0.115	0.092	0.169
	MAD2	Additive	0.428	0.362	0.361	0.110	0.092	0.169
		Additive+dominance	0.434	0.368	0.369	0.109	0.092	0.169
	MAD3	Additive	0.392	0.340	0.324	–	–	–
		Additive+dominance	0.427	0.358	0.350	–	–	–
	*P*_MA,MAD_	Additive	0.002	0.002	0.057	0.414	0.726	0.691
		Additive+dominance	<0.001	<0.001	<0.001	0.185	0.854	0.636
	*P*_MA, MAD2_	Additive	<0.001	<0.001	0.014	0.254	0.865	0.723
		Additive+dominance	<0.001	<0.001	<0.001	0.595	0.854	0.610

a MA = only additive effects included; MAD = additive and dominance (values) effects included; MAD2 = additive and dominance (deviations) effects included; and MAD3 = additive and dominance effects included as well as cows with genotype probabilities derived using genotyped ancestors.

### Largest SNP Effects

**Figure 1:**
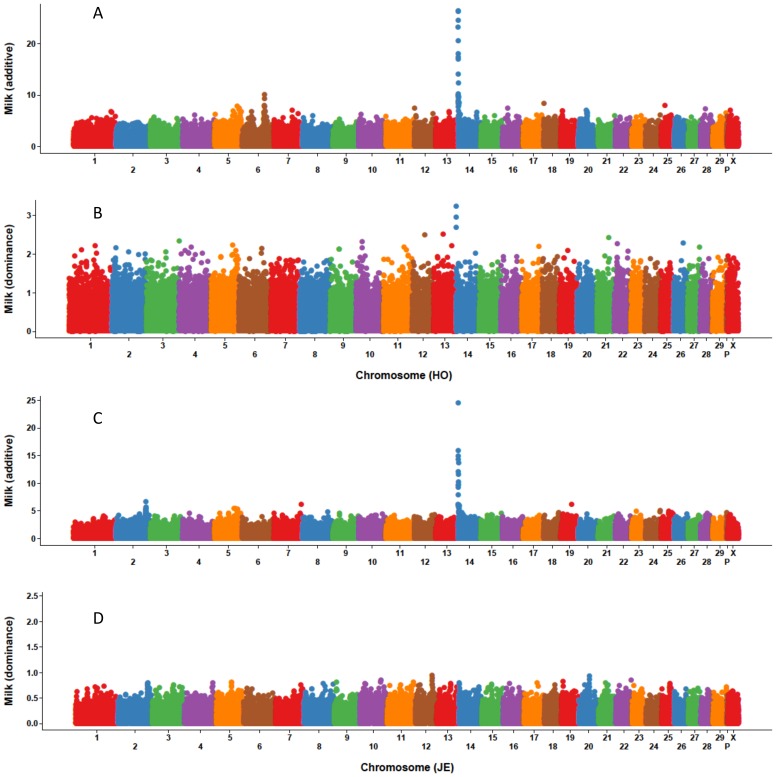
Size and location of marker additive and dominance effects for milk yield of Holsteins and Jerseys. Holstein additive (A) and dominance (B) effects and Jersey additive (C) and dominance (D) effects were estimated with a model that included additive and dominance (values) effects.

**Figure 2:**
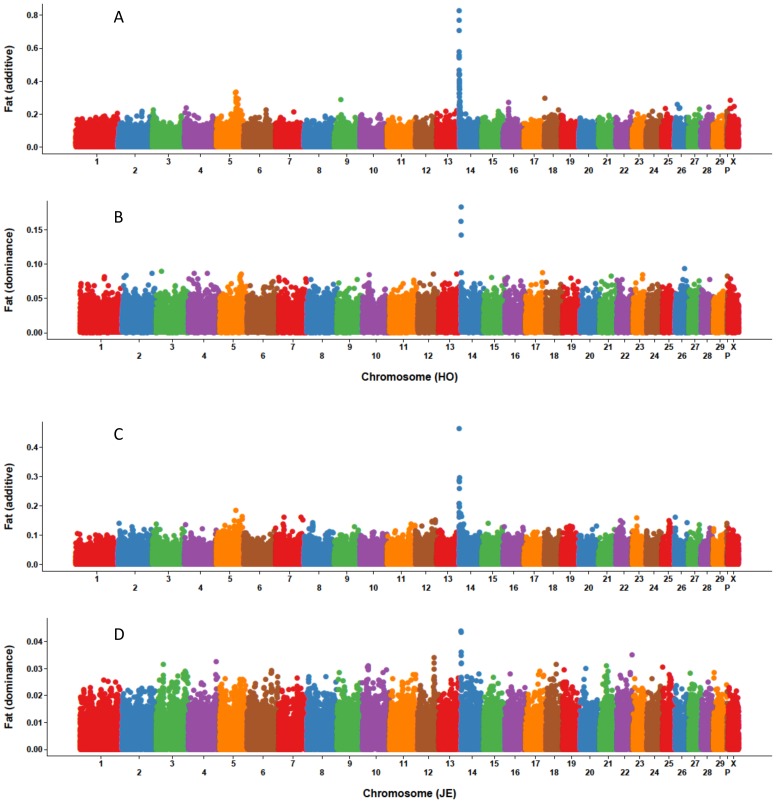
Size and location of marker additive and dominance effects for fat yield of Holsteins and Jerseys. Holstein additive (A) and dominance (B) effects and Jersey additive (C) and dominance (D) effects were estimated with a model that included additive and dominance (values) effects.

**Figure 3:**
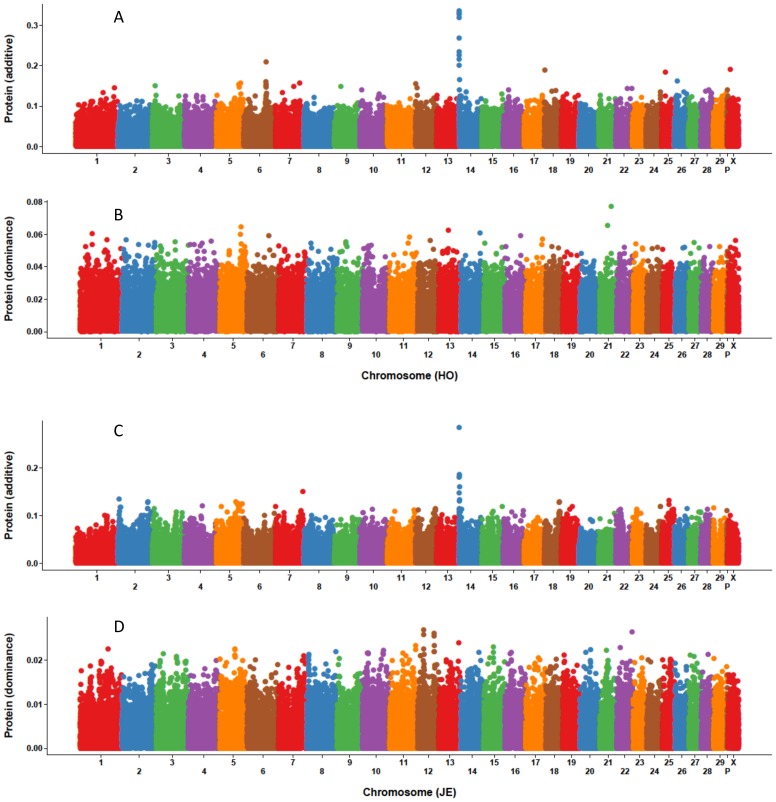
Size and location of marker additive and dominance effects for protein yield of Holsteins and Jerseys. Holstein additive (A) and dominance (B) effects and Jersey additive (C) and dominance (D) effects were estimated with a model that included additive and dominance (values) effects.

Based on additive and dominance SNP effects from MAD, Manhattan plots for eight traits were constructed, and the ten SNP with largest effect were characterized. Figures 1–3 show that the largest additive SNP effects are located on chromosome 14 near *DGAT1* [24] for all three yield traits for both breeds. For Holstein milk and fat yields as well as Jersey fat yield, the SNP with largest additive effect also had the largest dominance effect. The SNP effects for PL, DPR, SCS, fat% and protein% are not shown because the dominance effects were extremely small and the plots were not informative.

For yield traits, Table 7 lists the top 10 SNPs selected by dominance effects which were estimated using MAD; SNP locations are based on the UMD 3.1 assembly of the *Bos taurus* genome [25]. For both Holsteins and Jerseys, several SNPs on chromosome 14 had both large additive and dominance effects for fat yield. For Holsteins, three SNPs on chromosome 14 had large dominance and additive effects for both milk and fat yields. One SNP on chromosome 26 also had a large dominance effect for milk and fat yields, and chromosomes 13 and 21 each had one SNP with a large dominance effect for both milk and protein yields. No SNP had both large additive and dominance effects for Jersey milk or protein yield. For Jerseys, two SNPs on chromosome 12 and one SNP on chromosome 22 had a large dominance effect for all three yield traits; another SNP on chromosome 12 had a large dominance effect for both milk and protein yields.

**Table 7:** Characteristics of top ten single nucleotide polymorphisms for Holstein and Jersey milk, fat, and protein yields based on size of dominance effect from a model with additive and dominance (values) effects included.

Breed	Yield trait	SNP location^a^		SNP effect (BV kg)		Additive effect rank
		Chromosome	Position (base pairs)	Dominance	Additive	
Holstein	Milk	14	1,801,116	3.24	26.19	2
		14	1,651,311	2.96	26.39	1
		14	2,054,457	2.70	24.52	3
		13	40,525,851	2.52	0.46	34,006
		12	54,550,449	2.49	1.97	9,688
		21	47,902,442	2.42	2.05	8,936
		3	121,275,236	2.33	0.99	23,046
		10	21,865,155	2.33	0.34	36,692
		26	36,663,874	2.28	1.81	11,349
		22	10,376,267	2.26	0.17	40,871
	Fat	14	1,801,116	0.183	0.828	1
		14	1,651,311	0.163	0.769	2
		14	2,054,457	0.143	0.708	3
		26	36,663,874	0.094	0.048	16,041
		3	24,336,036	0.089	0.044	18,040
		14	2,117,455	0.088	0.541	7
		17	65,866,532	0.087	0.026	27,226
		4	23,648,508	0.087	0.004	42,123
		2	123,226,556	0.086	0.060	11,798
		4	74,397,417	0.086	0.038	20,782
	Protein	21	47,902,442	0.077	0.028	18,480
		21	32,811,807	0.065	0.058	4,693
		5	101,309,988	0.064	0.038	12,142
		13	40,525,851	0.062	0.019	25,662
		14	68,962,221	0.061	0.033	15,227
		1	47,856,002	0.060	0.028	18,242
		5	97,828,652	0.060	0.060	4,353
		6	86,643,810	0.059	0.109	217
		16	65,635,111	0.059	0.033	15,224
		11	75,760,570	0.058	0.083	1,206
Jersey	Milk	12	68,628,611	0.94	3.00	780
		20	39,691,234	0.92	1.50	7,930
		12	69,277,902	0.89	1.05	13,978
		20	39761822	0.87	1.68	6,171
		22	58,489,232	0.86	2.34	2,298
		10	84,516,867	0.85	1.46	8,346
		10	83,350,003	0.84	2.78	1,125
		12	68,678,678	0.83	3.26	506
		19	8,648,705	0.83	2.79	1,117
		9	9,396,200	0.82	0.34	30,510
	Fat	14	1,651,311	0.044	0.206	6
		14	1,696,470	0.043	0.206	7
		14	2,002,873	0.036	0.177	11
		22	58,489,232	0.035	0.055	4,540
		14	1,675,278	0.035	0.160	20
		12	68,628,611	0.034	0.144	33
		4	108,709,290	0.032	0.026	16,462
		12	68,678,678	0.032	0.147	32
		14	2,909,929	0.032	0.171	13
		14	2,217,163	0.032	0.282	4
	Protein	12	26,810,556	0.027	0.016	22,006
		22	58,489,232	0.026	0.065	1,405
		12	68,678,678	0.026	0.087	339
		12	26,883,203	0.026	0.016	21,301
		12	68,628,611	0.026	0.088	311
		13	77,572,954	0.024	0.041	6,175
		11	100,858,404	0.023	0.034	9,006
		12	69,277,902	0.023	0.055	2,727
		15	39,405,517	0.023	0.006	32,548
		22	12,470,007	0.023	0.101	122

a UMD 3.1 assembly of the *Bos taurus* genome [25].

Table 8 shows the top 10 SNPs selected by additive effect (from MAD) with SNPs on chromosome 14 excluded. No SNP had both large additive and dominance effects for either breed for any yield trait. Chromosome 5 had several SNP with a large additive effect for fat yield for both Jerseys and Holsteins. For milk yield, the SNP with the largest additive effects were on chromosomes 5, 6, 16, 18, and 25 for Holsteins and on chromosomes 2, 5, 7, and 19 for Jerseys. For protein yield, the SNP with the largest additive effects were on chromosomes 5, 6, 7, 12, 18, 25, 26, and X for Holsteins and on chromosomes 2, 5, 7, 18, and 25 for Jerseys. One SNP on chromosome 18 for Holsteins had a large additive effect for all three yield traits as did one SNP on chromosome 5 and another on chromosome 7 for Jerseys. Chromosome 16 for Holsteins had one SNP with a large additive effect for both milk and fat yields. Two SNPs on chromosome 6 and another on chromosome 25 for Holsteins had large additive effects for both milk and protein yields as did two SNPs on chromosome 2 and one SNP on chromosome 5 for Jerseys. The X chromosome for Holsteins had one SNP with a large additive effect for both fat and protein yields.

**Table 8:** Characteristics of top ten single nucleotide polymorphisms with chromosome 14 excluded for Holstein and Jersey milk, fat, and protein yields based on size of additive effect from a model with additive and dominance (values) effects included.

Breed	Yield trait	SNP location^a^		SNP effect (BV kg)		Dominance effect rank
		Chromosome	Position (base pairs)	Additive	Dominance	
Holstein	Milk	6	88,891,318	10.02	1.28	1,039
		6	88,592,295	9.28	2.03	29
		18	2,685,718	8.30	1.55	310
		6	88,069,548	7.94	1.12	1,957
		25	11,760,835	7.92	1.08	2,270
		5	105,870,613	7.87	0.01	42,548
		6	88,822,266	7.82	0.09	36,935
		6	88,656,290	7.68	0.55	12,298
		16	24,810,362	7.46	0.30	22,940
		5	112,775,479	7.46	1.63	235
	Fat	5	92,191,685	0.331	0.044	1,602
		5	88,776,643	0.329	0.004	35,959
		5	92,283,403	0.300	0.023	10,304
		18	2,685,718	0.295	0.073	53
		5	104,899,417	0.291	0.070	84
		9	27,356,886	0.286	0.018	14,877
		5	94,645,698	0.284	0.064	189
		5	92,618,397	0.282	0.021	12,098
		X	20,758,679	0.282	0.008	29,113
		16	24,810,362	0.272	0.005	34,244
	Protein	6	88,891,318	0.208	0.026	3,213
		X	20,758,679	0.191	0.006	28,602
		18	2,685,718	0.189	0.040	423
		25	11,760,835	0.184	0.030	1,913
		26	12,064,775	0.162	0.003	34,343
		6	87,222,751	0.160	0.009	20,708
		5	111,581,087	0.157	0.035	1,034
		7	95,577,863	0.156	0.006	28,129
		12	1,515,856	0.156	0.002	36,571
		6	88,656,290	0.154	0.018	8,392
Jersey	Milk	2	121,707,643	6.65	0.39	2,145
		19	42,425,855	6.21	0.61	210
		7	109,395,242	6.20	0.47	932
		2	122,707,290	5.64	0.71	71
		2	121,387,324	5.56	0.48	823
		5	90,752,558	5.43	0.36	2,805
		5	97,828,652	5.42	0.32	4,008
		2	121,476,153	5.36	0.45	1,166
		2	121,432,374	5.36	0.45	1,167
		2	121,649,725	5.26	0.46	1,014
	Fat	5	90,752,558	0.183	0.013	3,164
		5	117,133,270	0.162	0.024	168
		7	34,324,708	0.161	0.001	37,864
		7	103,779,001	0.161	0.006	13,556
		26	4,248,936	0.160	0.003	24,624
		23	19,308,267	0.158	0.000	38,693
		5	117,102,295	0.154	0.015	2,286
		12	86,426,256	0.152	0.000	39,802
		7	109,395,242	0.151	0.012	3,919
		5	84,702,280	0.149	0.002	28,164
	Protein	2	109,395,242	0.151	0.014	887
		25	3,950,794	0.135	0.006	9,175
		18	25,779,530	0.131	0.010	3,419
		5	56,397,946	0.129	0.009	4,848
		2	90,752,558	0.128	0.010	3,386
		18	121,707,643	0.128	0.010	3,067
		2	55,983,042	0.127	0.008	5,301
		5	121,387,324	0.127	0.016	453
		5	97,828,652	0.126	0.007	7,731
		2	105,664,687	0.125	0.006	9,426

a UMD 3.1 assembly of the *Bos taurus* genome [25].

## Discussion

The magnitude of dominance variance relative to phenotypic variance for different traits varied widely for genotyped Holstein and Jerseys cows in the United States. Dominance variances were larger for MAD than for MAD2. Dominance heritability from MAD for milk yield was 5% for Holsteins and 7% for Jerseys, which was slightly higher than the results reported by Sun et al. [11]. Result differences were caused by different models for estimating yield deviation and different methods for imputing missing genotypes, but the impact on Holstein results was smaller than for Jerseys because of the large Holstein data set. Few other studies have estimated dominance variance using Holstein genomic data. We verified that our software gives the same estimates of variance components and SNP effects as GVCBLUP [15] by comparing results when both were applied to the Jersey milk data and MAD2 model (see Text S1), but GVCBLUP cannot handle all the models we considered.

Additive and non-additive variances usually have been estimated using models with pedigree-based relationship matrices. Van Tassell et al. [26] estimated additive and dominance variance using Method R and reported results consistent with the findings of the current study for yield and SCS traits (5% and 1% dominance variance, respectively) but larger for PL (6%). For MAD, dominance variance relative to additive genetic variance was 18.9% for milk yield, 21.7% for fat yield, and 26.4% for protein yield for Holsteins and 21.7, 37.4, and 30.4%, respectively, for Jerseys. Misztal [27] reported a ratio of dominance to additive genetic variance of 17% for stature for U.S. Holsteins. However, Hoeschele et al. [28] reported ratios of 118% for days open and 161% for service period (days between first and last insemination) for U.S. Holsteins, and also showed that dominance variance changed significantly with slight differences in trait definition, e.g. at days open with an upper bound of 150 days, dominance heritability became very low. The change in estimates indicates some lack of precision, perhaps caused by solving for 3 genetic variances (A, D, and AA) in the same model, which also caused trouble in our study (results computed but not shown); furthermore different models (sire and maternal grandsire model vs animal model) and relationship matrices (pedigree vs genomic) as well as pre-selection (genotyped cows were offspring of genetically superior animals) all can lead to different results between our study with Hoeschele et al. [28]. In beef cattle, the ratio was >50% for weaning weight for Herefords, Gelbvieh, and Charolais [29], [30], and for post-weaning gain in Limousin beef cattle [31]. These results indicate that the range of estimates for non-additive genetic variance in different studies is large and may reflect different features of various traits and populations or large sampling error due to insufficient data. Fixed regression on inbreeding and heterosis accounted for effects of dominance on phenotypic mean in this study, and variance estimates accounted for additional covariances among relatives. The pre-adjusted phenotypes used in this study included inbreeding and heterosis effects, and an additional analysis (results not shown) on variance components estimation for Jersey indicated that removing inbreeding and heterosis effects from pre-adjusted phenotypes decreased dominance heritabilities slightly for yield traits (for example 7.0% vs. 5.9% for milk), but had very small effects on other traits (for example 1.2% vs. 1.1% for SCS). The inbreeding and heterosis effects in the model may account for changes in the mean rather than changes in the covariance among relatives.

The likelihood ratio test showed that a model with a dominance effect had better goodness of fit for yield traits than did a model with only an additive effect. Therefore, non-additive genetic variance is important for complex traits, and a model with non-additive genetic effects is expected to increase prediction accuracy. In this study, MAD was approximately 2% better than MA for predicting phenotypes in testing data sets. Lee et al. [8] predicted unobserved phenotypes using whole-genome SNP data and reported that the accuracy of prediction increased considerably when dominance effects were included compared with a purely additive genetic model. Their increased accuracy was 17% for coat color and 2% for percentage of CD8^+^ cells in mice; however, added epistasis did not contribute to accuracy. Su et al. [9] estimated additive and non-additive genetic variances and predicted genetic merit using genome-wide dense SNP; they found that reliabilities of genomic BV for animals without performance records increased 0.7 percentage points for a model that included additive and dominance effects compared with an additive-only model; the corresponding increase for a model that included additive and epistatic effects was only 0.3 percentage points.

The difference between MAD and MAD2 was how the dominance relationship matrix was calculated. In this study, estimates for dominance variance were larger and additive variances smaller for MAD compared with MAD2. Vitezica et al. [16] reported this same result for simulated data and concluded that MAD underestimates additive genetic variance and overestimates dominance variance; however, they did not compare the predictive ability of MAD and MAD2. In this study, MAD and MAD2 had no apparent difference in predictive ability, and the correlations between total genetic effects (or additive effects only) and phenotypes in testing data (or training data) were almost the same for the two models.

The MAD3 model was expected to increase predictive ability even more than MAD and MAD2 because it included sire-dam and sire-MGS groups to increase the available data; however, it did not. Perhaps because of the more complex model needed to deal with combined data (DATA_C_, DATA_S-D_, and DATA_S-MGS_), MAD3 underestimated additive heritability. A better model might treat the three groups as correlated phenotypes to account for differences in genotype accuracy and phenotype distributions between them. The cows with imputed genotype probabilities were offspring of genetically superior (elite) animals, and pre-selection may have affected the results and caused bias. Another issue that may need to be addressed is if including all of the genotyped females is optimal. Some elite cows were genomically tested after their phenotypes showed them to be superior and may represent only a small fraction of a herd (e.g., if a farmer tests only his five best animals). Such cows are highly selected, and predictions may become more accurate by limiting their data.

In addition to increased prediction accuracy, a model that includes additive and non-additive genetic effects could be beneficial for exploiting specific combining ability. Breeders should continue to select for additive merit but can also improve non-additive merit by considering interactions in mating programs [32]. Sun et al. [11] compared mating programs and found that expected progeny value for milk yield from linear programming using genomic relationship matrices increased 86 kg for Holsteins and 52 kg for Jerseys for the top 50 bulls for genomic BV for milk yield by including dominance effects. However, two practical limitations exist for implementing a model with both additive and non-additive genetic effects for genomic prediction [9]. First, the computational demand for models with both additive and non-additive genetic effects is generally high because both additive and non-additive genomic relationship matrices are dense, thus requiring greater computing resources or more efficient algorithms. The iteration-based SNP-BLUP used in this study greatly decreased the amount of memory needed and converged well for each of the three data groups, but it converged poorly for the combined data. Second, a reference population often consists of bulls that have records of progeny performance, and pseudo-observations (conventional EBV, de-regressed EBV, or means of corrected progeny performance) are commonly used as response variables. However, a genomic prediction model that includes non-additive genetic effects requires that the response variable is an individual record. Therefore, pseudo-observations are appropriate for an additive genetic model but not for a model that includes non-additive genetic effects.

The *DGAT1* gene is a major quantitative trait locus (QTL) on chromosome 14 that affects yield traits [24]. This study confirmed that the SNPs with the largest MAD additive effects were located on chromosome 14 for all three yield traits; those SNPs also had the largest dominance effects for fat yield for Holsteins and Jerseys as well as for Holstein milk yield. Boysen et al. [12] explored dominance effects using cow genotype probabilities based on bull genotypes and found significant (*P* ≤ 0.01) dominance effects for fat yield on chromosome 14 within the *DGAT1* region. The current study and Boysen et al. [12] both found no significant (*P* ≤ 0.01) dominance effects for SCS. A QTL that affects yield traits have been identified on chromosome 6 using granddaughter designs in U.S. [33], Dutch [34], and German [35] Holstein populations. In the current study, SNP on chromosome 6 had large additive effects for Holstein milk and protein yields. Cole et al. [36] studied the distribution and location of additive genetic effects for Holsteins using 5,285 bulls and confirmed the presence of two major genes for yield traits on chromosomes 6 and 14. Similar results also were reported by Cole et al. [37] using a population of genotyped U.S. Holstein cows. Wang et al. [38] performed a genome-wide association study for fat percentage in the German Holstein-Friesian population and uncovered a QTL region on chromosome 5. The current study also indentified a region on chromosome 5 with both large additive and dominance effects for Holstein yield traits.

## Conclusions

Dominance variance accounted for about 5 and 7% of total variance for yield traits for Holsteins and Jerseys, respectively, based on the MAD model. For PL, DPR, SCS, fat% and protein% dominance variances were very small, especially for Holsteins. The MAD model had smaller additive and larger dominance variance estimates compared with MAD2. The likelihood ratio test showed that a model with dominance effects included had better goodness of fit than an additive-only model for yield traits. Based on ten-fold cross-validation, the MAD and MAD2 models can increase prediction ability for Holstein and Jersey yield traits; improvements from the two models were similar. Prediction accuracy did not improve by including cows with derived genotypes. The largest additive effects were located on chromosome 14 for all three yield traits for both breeds, and those SNP also had the largest dominance effects for fat yield for Holsteins and Jerseys as well as Holstein milk yield. Dominance effects should be considered for inclusion in routine genomic evaluation models to improve prediction accuracy and exploit specific combining ability.

## Supporting Information

Text S1 (DOCX)
