# Supplementary material for: Improvement of Prediction Ability for Genomic Selection of Dairy Cattle by Including Dominance Effects
Source: PLoS One. 2014 Aug 1;9(8):e103934. doi: 10.1371/journal.pone.0103934 (PMC4118992; doi:10.1371/journal.pone.0103934)
Supplement: Text S1 — (DOCX) [file pone.0103934.s001.docx]

**Supplementary information**

To verify the formulas and theory implemented in our software, a numerical comparison with GVCBLUP [15] was made using the Jersey milk trait and MAD2 model.

To guarantee that both software used the same data and models we imported the additive and dominant covariance matrices generated by GVCBLUP into MMAP. The MMAP variance component estimates were identical to GVCBLUP. We then imported the MMAP variance estimates into our SNP-BLUP program to compute SNP effects. Additive breeding values and dominance deviations were computed from SNP effects following the GVCBLUP approach of dropping missing calls from the calculation. Our results were identical to GVCBLUP. Thus, our MAD2 model and MAD2_SNP_ models are equivalent.
